# Intranasal esketamine efficacy as a treatment for treatment-resistant depression, case series

**DOI:** 10.1192/j.eurpsy.2024.1440

**Published:** 2024-08-27

**Authors:** C. Delgado Marmisa, I. Álvarez Correa, H. Vizcaíno Herrezuelo, L. Egüen Recuero, E. Garrido Dobrito, L. Gayubo Moreo, B. Sanz-Aranguez, L. Delgado Tellez, L. Caballero Martínez, R. De Arce Cordón, B. Jiménez-Fernández

**Affiliations:** ^1^Psychiatry; ^2^Pharmacology, Hospital Universitario Puerta de Hierro Majadahonda, Majadahonda; ^3^Department of Psychiatry, Hospital Universitari Germans Trias i Pujol, Barcelona, Spain

## Abstract

**Introduction:**

Intranasal esketamine has been approved as a treatment for patients with treatment-resistant depression. We analyzed the results of its efficacy in 15 patients.

**Objectives:**

To evaluate the efficacy of intranasal Esketamine as a treatment in patients with treatment-resistant depression

**Methods:**

Case series

**Results:**

For the last 8 months, since the treatment with intranasal esketamine was approved for resistant depression, we have treated 14 patients with this drug. Through this process, we followed a standardized method consisting in the following steps:

On the first esketamine session (DAY 1) the patient has to fill a CGI and a MADRS scale.

On the second esketamine session (DAY 7) the patient has to fill a CGI, a MADRS scale, a form about the level of satisfaction with the drug and a last form in which they can include the secondary effects.

On week 6 since the start of the treatment, the patient has to fill again a CGI, a MADRS scale, a form about the level of satisfaction with the drug and a last form in which they can include the secondary effects.

In the 6th month since the start of the treatment, the patient has to fill again a CGI, a MADRS scale, a form about the level of satisfaction with the drug and a last form in which they can include the secondary effects they have perceived.

We analyzed and compared all of the previous data and obtained the following results:

At day 7: 64% of the patients had a response in the form of improvement, of which 66% were feeling “slightly better” and 33% were feeling “better”.

At week 6: 71% of the patients had a response in the form of improvement, of which 50% were feeling “slightly better” and the other 50% were feeling “better”.

At month 6: only 28% of the patients completed the treatment; of which 100% had a response in the form of improvement: 50% were feeling “slightly better”, 25% were feeling “better” and 25% were feeling “far better”.

**Conclusions:**

Although our data suggests that intranasal esketamine has been effective in short term depressive symptoms, we have yet no information about its medium and long-term efficacy or secondary effects. Nevertheless, other potential factors should be evaluated as they could affect the results in the long-term such as the difficulty in maintaining the treatment for more than 6 weeks.

In addition, the patients who experienced the most improvement according to our data were patients with a TAB diagnosis, so this could be an interesting research focus.

**Disclosure of Interest:**

None Declared

